# Redetermination of 1,4-dimethoxy­benzene

**DOI:** 10.1107/S1600536808044231

**Published:** 2009-01-08

**Authors:** Robbie Iuliucci, Cody L. Hoop, Atta M. Arif, James K. Harper, Ronald J. Pugmire, David M. Grant

**Affiliations:** a Department of Chemistry, Washington and Jefferson College, 60 South Lincoln Street, Washington, PA 15301, USA; b Department of Chemistry, University of Utah, Salt Lake City, UT 84112, USA

## Abstract

The structure of the centrosymmetric title compound, C_8_H_10_O_2_, originally determined by Goodwin *et al.* [*Acta Cryst.*(1950), **3**, 279–284], has been redetermined to modern standards of precision to aid in its use as a model compound for ^13^C chemical-shift tensor measurements in single-crystal NMR studies. In the crystal structure, a C—H⋯O inter­action helps to establish the packing.

## Related literature

For previous structural studies of the title compound, see: Goodwin *et al.* (1950 ▶); Carter *et al.* (1988 ▶).
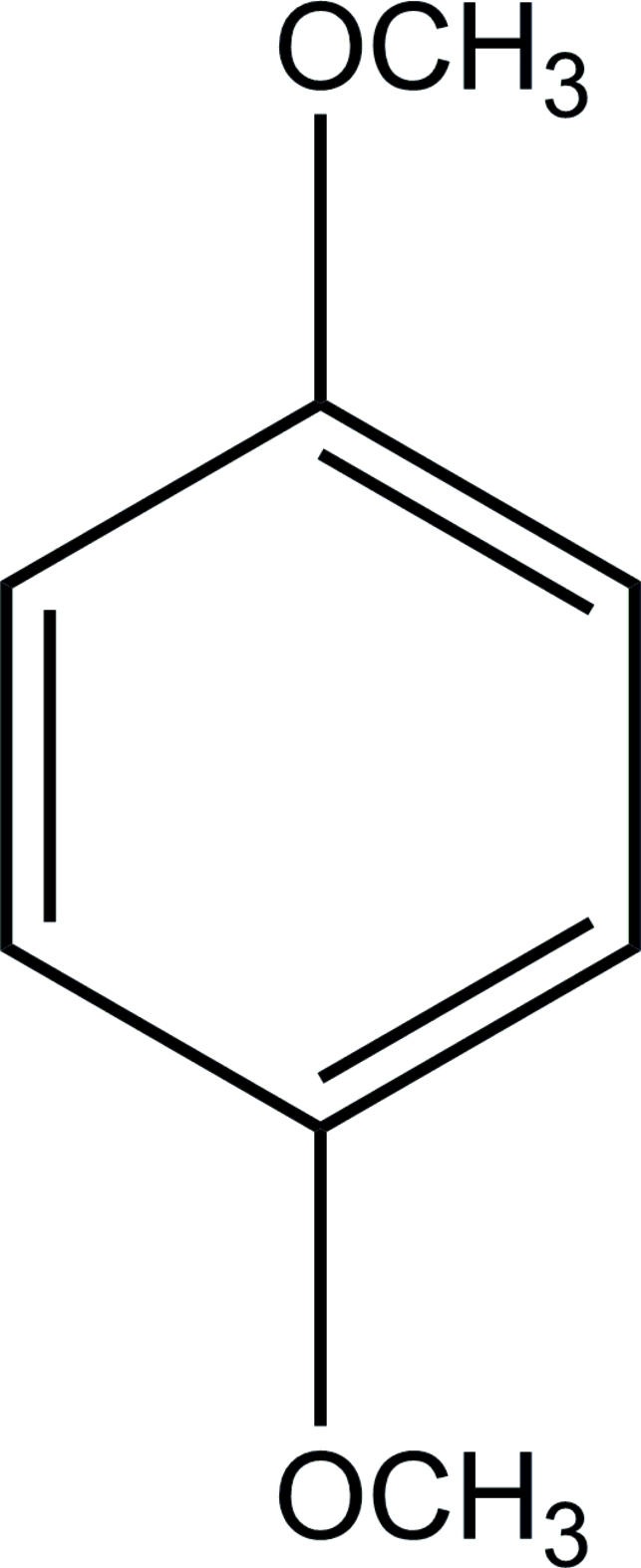


## Experimental

### 

#### Crystal data

C_8_H_10_O_2_
*M* *_r_* = 138.16
Orthorhombic, 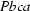

*a* = 7.1757 (3) Å
*b* = 6.2769 (2) Å
*c* = 16.5573 (7) Å
*V* = 745.76 (5) Å^3^
*Z* = 4
Mo *K*α radiation
μ = 0.09 mm^−1^
*T* = 150 (1) K
0.33 × 0.30 × 0.23 mm

#### Data collection

Nonius KappaCCD diffractometer
Absorption correction: multi-scan (*DENZO–SMN*; Otwinowski & Minor, 1997 ▶) *T*_min_ = 0.972, *T*_max_ = 0.980
1510 measured reflections
847 independent reflections
732 reflections with *I* > 2σ(*I*)
*R*_int_ = 0.013

#### Refinement

*R*[*F* ^2^ > 2σ(*F* ^2^)] = 0.037
*wR*(*F* ^2^) = 0.101
*S* = 1.09
847 reflections
66 parameters
All H-atom parameters refined
Δρ_max_ = 0.19 e Å^−3^
Δρ_min_ = −0.16 e Å^−3^

### 

Data collection: *COLLECT* (Hooft, 1998 ▶); cell refinement: *DENZO–SMN* (Otwinowski & Minor, 1997 ▶); data reduction: *DENZO–SMN*; program(s) used to solve structure: *SIR97* (Altomare *et al.*, 1999 ▶); program(s) used to refine structure: *SHELXL97* (Sheldrick, 2008 ▶); molecular graphics: *WinGX* (Farrugia, 1999 ▶) and *ORTEP-3* (Farrugia, 1997 ▶); software used to prepare material for publication: *publCIF* (Westrip, 2009 ▶).

## Supplementary Material

Crystal structure: contains datablocks I, global. DOI: 10.1107/S1600536808044231/hb2878sup1.cif

Structure factors: contains datablocks I. DOI: 10.1107/S1600536808044231/hb2878Isup2.hkl

Additional supplementary materials:  crystallographic information; 3D view; checkCIF report

**Fig. 1.**
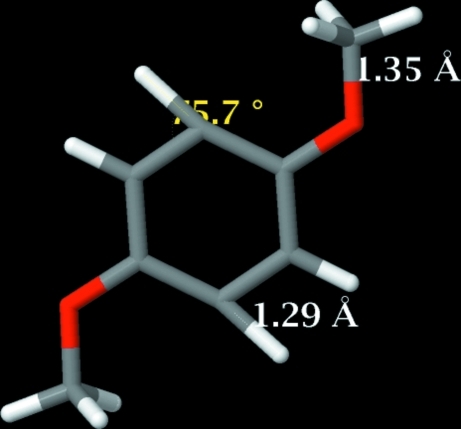
The structure of (I) according to Goodwin et al. (1950).

Enhanced figure: interactive version of Fig. 1

**Fig. 2.**
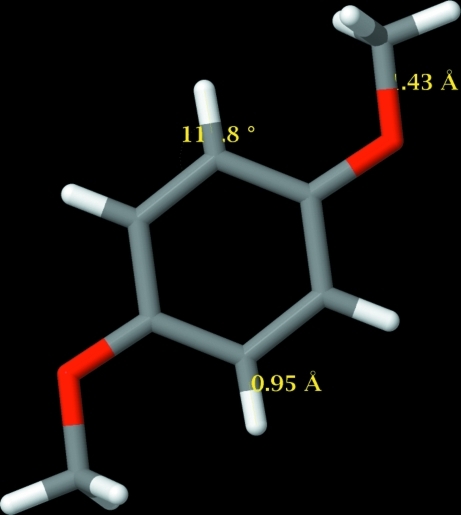
The redetermined structure of (I) from the present study.

Enhanced figure: interactive version of Fig. 2

## Figures and Tables

**Table 1:** Hydrogen-bond geometry (Å, °)

*D*—H⋯*A*	*D*—H	H⋯*A*	*D*⋯*A*	*D*—H⋯*A*
C4—H4*A*⋯O1^i^	1.019 (16)	2.552 (15)	3.4381 (15)	145.1 (10)

Symmetry code: (i) 
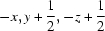
.

## supplementary crystallographic information

### Comment

Large single-crystals of organic compounds can be challenging to grow. Substituted methoxybenzenes are one exception and single-crystals on the order of centimeters can be obtained. The ease of crystal growth has enabled substituted methoxybenzenes to be studied by single-crystal NMR experiments. Pioneering work on the development of the two-dimensional single-crystal chemical-shift chemical-shift correlation NMR experiments utilized large crystals of 1,4-dimethoxybenzene (Carter *et al.*, 1988). In 1950 Goodwin *et al.* obtained the first X-ray diffraction structure for 1,4-dimethoxybenzene. This structure (*R*-factor = 0.12) is shown in Fig. 1 and reported an unusual H–C–C angle of 75.7°, which prompted the acquisition of a second structure (Carter *et al.*, 1988). More typical H–C–C angles were observed with this new refinement and this structure (*R*-factor = 0.067) was used to assign tensor orientations in the single-crystal NMR analysis. Inadvertently, the second structure was never submitted to the Cambridge Crystallographic database. Here, the acquisition of a third structure is reported to correct this oversight. The new structure (*R*-factor = 0.038) is shown in Fig. 2. The unit-cell and space group of the previous studies are confirmed.

Acquisition of this third, more accurate, structure is beneficial to NMR studies because the ^13^C chemical shift tensor data of 1,4-dimethoxybenzene continue to serve as a standard to evaluate new chemical-shift tensor measurement methods as well as to assess electronic structure methods for computing magnetic properties of molecules.

### Refinement

The H atoms were located in difference maps and their positions and U_iso_ values were freely refined.

### Crystal data

**Table:**

C_8_H_10_O_2_	*D*_x_ = 1.231 Mg m^−^^3^
*M**_r_* = 138.16	Melting point: 329 K
Orthorhombic, *P**b**c**a*	Mo *K*α radiation, λ = 0.71073 Å
Hall symbol: -P 2ac 2ab	Cell parameters from 13221 reflections
*a* = 7.1757 (3) Å	θ = 1.0–27.5°
*b* = 6.2769 (2) Å	µ = 0.09 mm^−^^1^
*c* = 16.5573 (7) Å	*T* = 150 K
*V* = 745.76 (5) Å^3^	Prism, colorless
*Z* = 4	0.33 × 0.30 × 0.23 mm
*F*(000) = 296	

### Data collection

**Table:**

Nonius KappaCCD diffractometer	847 independent reflections
Radiation source: fine-focus sealed tube	732 reflections with *I* > 2σ(*I*)
graphite	*R*_int_ = 0.013
φ and ω scans	θ_max_ = 27.5°, θ_min_ = 3.8°
Absorption correction: multi-scan (*DENZO*–SMN; Otwinowski & Minor, 1997)	*h* = −9→9
*T*_min_ = 0.972, *T*_max_ = 0.980	*k* = −8→8
1510 measured reflections	*l* = −21→21

### Refinement

**Table:**

Refinement on *F*^2^	Primary atom site location: structure-invariant direct methods
Least-squares matrix: full	Secondary atom site location: difference Fourier map
*R*[*F*^2^ > 2σ(*F*^2^)] = 0.037	Hydrogen site location: difference Fourier map
*wR*(*F*^2^) = 0.101	All H-atom parameters refined
*S* = 1.09	*w* = 1/[σ^2^(*F*_o_^2^) + (0.0517*P*)^2^ + 0.1487*P*] where *P* = (*F*_o_^2^ + 2*F*_c_^2^)/3
847 reflections	(Δ/σ)_max_ < 0.001
66 parameters	Δρ_max_ = 0.19 e Å^−^^3^
0 restraints	Δρ_min_ = −0.16 e Å^−^^3^
none constraints	

### Special details

**Table:**

Experimental. The program *DENZO-SMN* (Otwinowski & Minor, 1997) uses a scaling algorithm (Fox & Holmes, 1966) which effectively corrects for absorption effects. High redundancy data were used in the scaling program hence the 'multi-scan' code word was used. No transmission coefficients are available from the program (only scale factors for each frame). The scale factors in the experimental table are calculated from the 'size' command in the *SHELXL97* input file.
Geometry. All e.s.d.'s (except the e.s.d. in the dihedral angle between two l.s. planes) are estimated using the full covariance matrix. The cell e.s.d.'s are taken into account individually in the estimation of e.s.d.'s in distances, angles and torsion angles; correlations between e.s.d.'s in cell parameters are only used when they are defined by crystal symmetry. An approximate (isotropic) treatment of cell e.s.d.'s is used for estimating e.s.d.'s involving l.s. planes.
Refinement. Refinement of *F*^2^ against ALL reflections. The weighted *R*-factor *wR* and goodness of fit *S* are based on *F*^2^, conventional *R*-factors *R* are based on *F*, with *F* set to zero for negative *F*^2^. The threshold expression of *F*^2^ > 2σ(*F*^2^) is used only for calculating *R*-factors(gt) *etc*. and is not relevant to the choice of reflections for refinement. *R*-factors based on *F*^2^ are statistically about twice as large as those based on *F*, and *R*- factors based on ALL data will be even larger.

### Fractional atomic coordinates and isotropic or equivalent isotropic displacement parameters (Å^2^)

**Table:**

	*x*	*y*	*z*	*U*_iso_*/*U*_eq_	
O1	0.01305 (11)	0.15781 (13)	0.15601 (4)	0.0315 (3)	
C2	0.09537 (13)	−0.10269 (16)	0.06126 (6)	0.0257 (3)	
C3	−0.09472 (14)	0.18955 (17)	0.01604 (6)	0.0264 (3)	
C1	0.00160 (12)	0.08587 (17)	0.07767 (6)	0.0243 (3)	
C4	−0.0849 (2)	0.3490 (2)	0.17534 (8)	0.0410 (3)	
H2	0.1619 (17)	−0.175 (2)	0.1048 (8)	0.033 (3)*	
H3	−0.1604 (18)	0.319 (2)	0.0253 (7)	0.031 (3)*	
H4A	−0.061 (2)	0.375 (2)	0.2352 (10)	0.050 (4)*	
H4B	−0.221 (3)	0.323 (2)	0.1672 (9)	0.057 (5)*	
H4C	−0.036 (2)	0.470 (3)	0.1413 (11)	0.056 (4)*	

### Atomic displacement parameters (Å^2^)

**Table:**

	*U*^11^	*U*^22^	*U*^33^	*U*^12^	*U*^13^	*U*^23^
O1	0.0387 (5)	0.0328 (5)	0.0231 (4)	0.0040 (3)	−0.0018 (3)	−0.0031 (3)
C2	0.0249 (5)	0.0268 (5)	0.0255 (5)	0.0015 (4)	−0.0017 (4)	0.0043 (4)
C3	0.0259 (5)	0.0244 (5)	0.0288 (6)	0.0029 (4)	0.0012 (4)	0.0014 (4)
C1	0.0241 (5)	0.0267 (5)	0.0220 (5)	−0.0028 (4)	0.0012 (3)	0.0007 (4)
C4	0.0534 (8)	0.0377 (7)	0.0321 (6)	0.0092 (6)	−0.0004 (5)	−0.0104 (5)

### Geometric parameters (Å, °)

**Table:**

O1—C1	1.3759 (12)	C3—C1	1.3937 (14)
O1—C4	1.4269 (14)	C3—H3	0.953 (13)
C2—C1	1.3883 (15)	C4—H4A	1.020 (16)
C2—C3^i^	1.3912 (15)	C4—H4B	0.998 (18)
C2—H2	0.977 (13)	C4—H4C	1.010 (18)
C3—C2^i^	1.3912 (15)		
			
C1—O1—C4	117.26 (9)	O1—C1—C3	124.51 (10)
C1—C2—C3^i^	120.83 (9)	C2—C1—C3	119.68 (10)
C1—C2—H2	119.4 (7)	O1—C4—H4A	105.8 (8)
C3^i^—C2—H2	119.8 (7)	O1—C4—H4B	108.4 (9)
C2^i^—C3—C1	119.48 (10)	H4A—C4—H4B	108.8 (12)
C2^i^—C3—H3	118.7 (8)	O1—C4—H4C	109.7 (9)
C1—C3—H3	121.8 (8)	H4A—C4—H4C	111.2 (12)
O1—C1—C2	115.81 (9)	H4B—C4—H4C	112.6 (12)
			
C4—O1—C1—C2	−178.76 (10)	C3^i^—C2—C1—C3	0.10 (16)
C4—O1—C1—C3	1.69 (15)	C2^i^—C3—C1—O1	179.44 (9)
C3^i^—C2—C1—O1	−179.47 (9)	C2^i^—C3—C1—C2	−0.10 (16)

Symmetry codes: (i) −*x*, −*y*, −*z*.

### Hydrogen-bond geometry (Å, °)

**Table:**

*D*—H···*A*	*D*—H	H···*A*	*D*···*A*	*D*—H···*A*
C4—H4A···O1^ii^	1.019 (16)	2.552 (15)	3.4381 (15)	145.1 (10)

Symmetry codes: (ii) −*x*, *y*+1/2, −*z*+1/2.

## References

[bb1] Altomare, A., Burla, M. C., Camalli, M., Cascarano, G. L., Giacovazzo, C., Guagliardi, A., Moliterni, A. G. G., Polidori, G. & Spagna, R. (1999). *J. Appl. Cryst.* **32**, 115–119.

[bb2] Carter, C. M., Facelli, J. C., Alderman, D. W., Grant, D. M., Dalley, N. K. & Wilson, B. E. (1988). *J. Chem. Soc. Faraday Trans. 1*, **84**, 3673–3690.

[bb3] Farrugia, L. J. (1997). *J. Appl. Cryst.* **30**, 565.

[bb4] Farrugia, L. J. (1999). *J. Appl. Cryst.* **32**, 837–838.

[bb5] Goodwin, T. H., Przybylska, M. & Robertson, J. M. (1950). *Acta Cryst.* **3**, 279–284.

[bb6] Hooft, R. W. W. (1998). *COLLECT* Nonius BV, Delft, The Netherlands.

[bb7] Otwinowski, Z. & Minor, W. (1997). *Methods in Enzymology*, Vol. 276, *Macromolecular Crystallography*, Part A, edited by C. W. Carter Jr & R. M. Sweet, pp. 307–326. New York: Academic Press.

[bb8] Sheldrick, G. M. (2008). *Acta Cryst.* A**64**, 112–122. 10.1107/S0108767307043930 18156677

[bb9] Westrip, S. P. (2009). *publCIF. * In preparation.

